# The Latest Treatment for Eclampsia

**Published:** 1914-05-09

**Authors:** 


### The Latest Treatment for Eclampsia.

The doubts and mists which obscure the true pathology of eclampsia and render its treatment so largely empirical are well known. In America, as we have noted before, much more strenuous efforts are being made to clear them away than are displayed by British obstetricians. The latest hypothesis is ingenious, if rather far-fetched. It regards the breasts, not the uterus, placenta, fœtus, or kidney, as the prime source of toxins which set up eclampsia. This idea was evolved from the study of a disease of cattle known as parturient paresis, which presents certain analogies with eclampsia, and is attributed, with some apparent justice, to a mammary origin. Hence the suggestion that mammary toxins are at the bottom of eclampsia, and that it may be possible to cure the latter by measures similar to those already shown to be efficacious for parturient paresis. This consists in the injection of air, or oxygen, into the breast, with the object of distending it so as to diminish the circulation of blood through it, and so hindering both the formation and the absorption of the toxins. Several Continental surgeons have tried this, and some apparent successes have been reported. But the obvious criticism that such injections are very unlikely to form any perceptible hindrance to the circulation was soon perceived, and sundry thorough-going German clinicians promptly met the objection by proposing and carrying out immediate amputation of both breasts in serious cases of eclampsia. Prentiss Wilson contributes to the *American Journal of Obstetrics and Gynæcology* a thoughtful review of the subject, with details of the published cases, twenty-nine in number, in which one or other of these forms of treatment has been employed. He admits the striking similarities between parturient paresis and eclampsia, but notes the following differences: Parturient paresis rarely attacks primiparous animals, whereas primiparity markedly disposes to eclampsia. Parturient paresis occurs almost entirely post-partum; eclampsia shows no special predilection for this period. Sugar is an almost constant ingredient of the urine in parturient paresis, but is rarely found in eclamptic urine. He proceeds to the conclusion that the mammary theory of eclampsia is probably merely specious, but deserves careful and thorough investigation; especially the oxygen injection treatment, which, at any rate, is harmless, should be practically tested.

